# Modulation of the expression of genes related to the system of amyloid-beta metabolism in the brain as a novel mechanism of ceftriaxone neuroprotective properties

**DOI:** 10.1186/s12868-018-0412-5

**Published:** 2018-04-19

**Authors:** Maria A. Tikhonova, Tamara G. Amstislavskaya, Victor M. Belichenko, Larisa A. Fedoseeva, Sergey P. Kovalenko, Ekaterina E. Pisareva, Alla S. Avdeeva, Nataliya G. Kolosova, Nikolai D. Belyaev, Lyubomir I. Aftanas

**Affiliations:** 1 Federal State Budgetary Scientific Institution “Scientific Research Institute of Physiology and Basic Medicine” (SRIPhBM), Timakov St., 4, Novosibirsk, 630117 Russia; 2 Novosibirsk State University, Novosibirsk, 630090 Russia; 3 Federal Research Center “Institute of Cytology and Genetics”, Siberian Branch of the Russian Academy of Sciences, Novosibirsk, 630090 Russia; 4 University of Leeds, Leeds, LS2 9JT UK

**Keywords:** Ceftriaxone, Neuroprotection, Alzheimer’s disease, Amyloid metabolism, Gene expression, mRNA, Rats

## Abstract

**Background:**

The dominant hypothesis about the pathogenesis of Alzheimer’s disease (AD) is the “amyloid cascade” concept and modulating the expression of proteins involved in the metabolism of amyloid-beta (Aβ) is proposed as an effective strategy for the prevention and therapy of AD. Recently, we found that an antibiotic ceftriaxone (CEF), which possesses neuroprotective activity, reduced cognitive deficits and neurodegenerative changes in OXYS rats, a model of sporadic AD. The molecular mechanisms of this effect are not completely clear, we suggested that the drug might serve as the regulator of the expression of the genes involved in the metabolism of Aβ and the pathogenesis of AD. The study was aimed to determine the effects of CEF on mRNA levels of *Bace1* (encoding β-secretase BACE1 involved in Aβ production), *Mme*, *Ide*, *Ece1*, *Ace2* (encoding enzymes involved in Aβ degradation), *Epo* (encoding erythropoietin related to endothelial function and clearance of Aβ across the blood brain barrier) in the frontal cortex, hippocampus, striatum, hypothalamus, and amygdala of OXYS and Wistar (control strain) male rats. Starting from the age of 14 weeks, animals received CEF (100 mg/kg/day, i.p., 36 days) or saline. mRNA levels were evaluated with RT-qPCR method. Biochemical parameters of plasma were measured for control of system effects of the treatment.

**Results:**

To better understand strain variations studied here, we compared the gene expression between untreated OXYS and Wistar rats. This comparison showed a significant decrease in mRNA levels of *Ace2* in the frontal cortex and hypothalamus, and of *Actb* in the amygdala of untreated OXYS rats. Analysis of potential effects of CEF revealed its novel targets. In the compound-treated OXYS cohort, CEF diminished mRNA levels of *Bace1* and *Ace2* in the hypothalamus, and *Aktb* in the frontal cortex. Furthermore, CEF augmented *Mme*, *Ide*, and *Epo* mRNA levels in the amygdala as well as the levels of *Ece1* and *Aktb* in the striatum. Finally, CEF also attenuated the activity of ALT and AST in plasma of OXYS rats.

**Conclusion:**

Those findings disclosed novel targets for CEF action that might be involved into neuroprotective mechanisms at early, pre-plaque stages of AD-like pathology development.

## Background

Alzheimer’s disease (AD) is one of the most common neurodegenerative disorders of the central nervous system. Loss of neurons in multiple brain regions (mainly in the cortical and subcortical areas and hippocampus) leads to cognitive dysfunctions including memory impairment as well as to behavioral and physiological changes. The dominant hypothesis of the pathogenesis of AD is the “amyloid cascade” concept. The excessive accumulation and aggregation of amyloid-beta (Aβ) in AD is assigned as a key role in triggering the chain of pathological neurodegenerative processes [1, 2]. Major components of the Aβ deposits are 38–43 amino acids-long fragments derived from proteolytic processing of the amyloid precursor protein (APP) [3]. Especially Aβ42 elevation is related to AD progression due to its high tendency to aggregate. Oligomeric Aβ species represent the most toxic forms causing impaired synaptic and neuronal functions [4, 5].

The Aβ levels in the brain depend on its de novo formation by amyloidogenic APP processing with β-secretase BACE1 and also on its elimination via different mechanisms including its proteolytic degradation, transport processes, cell mediated clearance, and its deposition into insoluble aggregates [2, 6]. It is believed that the imbalance between the Aβ production and removal contributes to the abnormal Aβ accumulation, and those mechanisms become impaired with aging and disease [7]. Hence, modulating the expression of proteins involved in the metabolism of Aβ has attracted increasing attention and is regarded as quite a promising strategy for AD therapy by lowering Aβ deposition in the brain [6, 8]. It should be noted that there is no one specific way of Aβ catabolism. A range of the potential amyloid-degrading proteolytic enzymes have been identified that hydrolyse a range of other physiological substrates as well. In the present study we focused on those enzymes that showed a connection with AD and Aβ degrading activity in vivo, including neprilysin (NEP) peptidase that is considered as a major Aβ degrading enzyme in the brain [8, 9].

OXYS rat strain with hereditary defined accelerated senescence was produced in the Institute of Cytology and Genetics SB RAS (Novosibirsk, Russia) with selective breeding of Wistar rats that were highly sensitive to cataractogenic effect of d-galactose. The OXYS rats are known for shortened lifespan and an early development of age-related pathological phenotypes [10]. Recently found signs of early neurodegeneration, cognitive decline, Aβ deposits, and increased tau phosphorylation in rats of the OXYS strain [11–14] along with the absence of specific for the early familial forms of AD mutations in *App*, *Psen1*, and *Psen2* genes in the OXYS genome [12] lend themselves to be a faithful model of sporadic AD.

Recently, we found that an antibiotic ceftriaxone (CEF), which possesses neuroprotective activity, reduced cognitive and neuronal deficits in OXYS rats [15]. The molecular mechanisms of this effect are not completely clear, therefore suggest that the drug might serve as the regulator of the gene expression involved in the metabolism of Aβ and the pathogenesis of AD. Hence, the present study was aimed to determine mRNA levels of *Bace1* (gene of β-secretase BACE1 involved in Aβ production), *Mme*, *Ece1*, *Ide*, *Ace2* (genes of enzymes involved in Aβ degradation), and *Epo* (gene for erythropoietin related to endothelial function and clearance of Aβ across the blood brain barrier) in the frontal cortex, hippocampus, striatum, hypothalamus, and amygdala of Wistar and OXYS rats as well as the potential effects of CEF on their modulation.

## Results

### Analysis of mRNA levels of CEF target genes in the amygdala

**Fig. 1:**
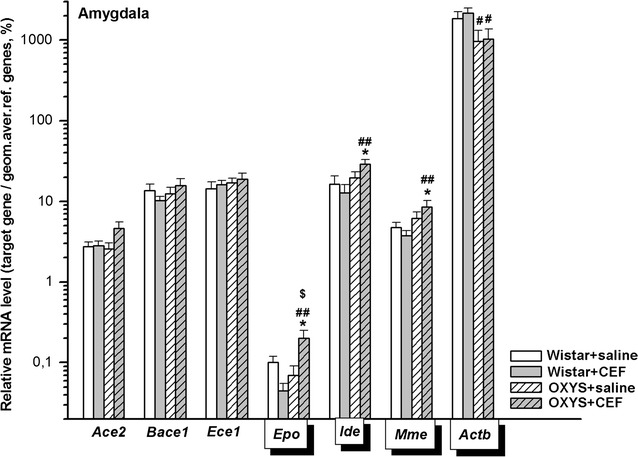
Effects of CEF on mRNA levels of *Ace2*, *Bace1*, *Ece1*, *Epo*, *Ide*, *Mme*, and *Actb* in the amygdala of OXYS and Wistar rats. The data represent the relative mRNA levels of target genes to geometrical mean of *B2m*, *Hprt1*, and *Mapk6* mRNA levels. The data are expressed as the Mean ± S.E.M. of the values obtained in an independent group of animals (*n* = 7–10 per group). Statistically significant differences: *P < 0.05 versus “Wistar + saline” group; ^#^*P* < 0.05; ^##^*P* < 0.01 versus “Wistar + CEF” group; ^$^*P* < 0.05 versus “OXYS + saline” group

 Abundance of mRNA species in the amygdala is summarized in Fig. 1.

#### *Ace2*

According to two-way ANOVA, there was a tendency in influence of the “Ceftriaxone” factor [F(1, 32) = 3.167, *P* = 0.085] and no significant effect of the “Genotype” factor [F(1, 32) = 1.851, *P* > 0.05] or of the interaction between the factors [F(1, 32) = 2.653, *P* > 0.05] on the mRNA levels of *Ace2* in the amygdala of the rats.

#### *Bace1*

Two-way ANOVA did not show a significant effect of the “Genotype” [F(1, 32) < 1] or “Ceftriaxone” [F(1, 32) < 1] factor as well as of the interaction between the factors [F(1, 32) = 1.517, *P* > 0.05] on mRNA levels of *Bace1*.

#### *Ece1*

Two-way ANOVA did not show a significant effect of the “Genotype” [F(1, 34) < 1] or “Ceftriaxone” [F(1, 34) < 1] factor as well as of the interaction between the factors [F(1, 34) < 1] on the mRNA levels of *Ece1*.

#### *Epo*

According to two-way ANOVA, there was a significant influence of the “Genotype” factor [F(1, 22) = 4.314, *P* < 0.05] and of the interaction between the factors [F(1, 22) = 8.383, *P* < 0.01] but no significant effect of the “Ceftriaxone” factor [F(1, 22) = 1.452, *P* > 0.05] on the mRNA levels of *Epo* in the amygdala of the rats. LSD post hoc test revealed that the parameter was significantly higher in the rats of “OXYS + CEF” group versus that in Wistar rats of “Wistar + saline” (*P* < 0.05) and “Wistar + CEF” (*P* < 0.01) or “OXYS + saline” (*P* < 0.05) group.

#### *Ide*

According to two-way ANOVA, there was a significant influence of the “Genotype” factor [F(1, 32) = 6.163, *P* < 0.05], a tendency for the interaction between the factors [F(1, 32) = 2.935, *P* = 0.096] and no significant effect for the “Ceftriaxone” factor [F(1, 32) < 1] on the mRNA levels of *Ide* in the amygdala of the rats. LSD post hoc test revealed that the parameter was significantly higher in the rats of “OXYS + CEF” group versus that in Wistar rats of “Wistar + saline” (*P* < 0.05) or “Wistar + CEF” (*P* < 0.01) group.

#### *Mme*

According to two-way ANOVA, there was a significant influence of the “Genotype” factor [F(1, 31) = 7.488, *P* < 0.05], and no significant effect for the “Ceftriaxone” factor [F(1, 31) < 1] or for the interaction between the factors [F(1, 31) = 2.255, *P* > 0.05] on the mRNA levels of *Mme* in the amygdala of the rats. LSD post hoc test revealed that the parameter was significantly higher in the rats of “OXYS + CEF” group versus that in Wistar rats of “Wistar + saline” (*P* < 0.05) or “Wistar + CEF” (*P* < 0.01) group.

#### *Actb*

According to two-way ANOVA, there was a significant influence of the “Genotype” factor [F(1, 31) = 7.610, *P* < 0.01], and no significant effect for the “Ceftriaxone” factor [F(1, 31) < 1] or for the interaction between the factors [F(1, 31) < 1] on the mRNA levels of *Actb* in the amygdala of the rats. LSD post hoc test revealed that the parameter was significantly higher in the rats of “Wistar + CEF” group versus that in OXYS rats of “OXYS + saline” (*P* < 0.05) or “OXYS + CEF” (*P* < 0.05) group.

### Analysis of mRNA levels of CEF target genes in the hypothalamus

**Fig. 2:**
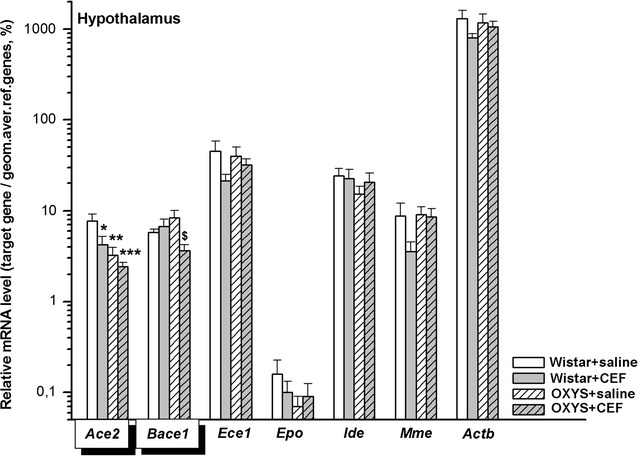
Effects of CEF on mRNA levels of *Ace2*, *Bace1*, *Ece1*, *Epo*, *Ide*, *Mme*, and *Actb* in the hypothalamus of OXYS and Wistar rats. The data represent the relative mRNA levels of target genes to geometrical mean of *B2m*, *Hprt1*, and *Mapk6* mRNA levels. The data are expressed as the Mean ± S.E.M. of the values obtained in an independent group of animals (*n* = 7–10 per group). Statistically significant differences: *P < 0.05; **P < 0.01; ***P < 0.001 versus “Wistar + saline” group; ^$^*P* < 0.05 versus “OXYS + saline” group

The results on the mRNA abundance in the hypothalamus are summarized in Fig. 2.

#### *Ace2*

According to two-way ANOVA, there were a significant influence of the “Genotype” factor [F(1, 28) = 10.643, *P* < 0.01] and of the “Ceftriaxone” factor [F(1, 28) = 4.955, *P* < 0.05] but no significant effect of the interaction between the factors [F(1, 28) = 1.973, *P* > 0.05] on the mRNA levels of *Ace2* in the hypothalamus of the rats. LSD post hoc test revealed that the parameter was significantly higher in the rats of “Wistar + saline” group versus that in rats of “Wistar + CEF” (*P* < 0.05) and “OXYS + saline” (*P* < 0.01) or “OXYS + CEF” (*P* < 0.001) group.

#### *Bace1*

According to two-way ANOVA, there was a significant influence of the interaction between the factors [F(1, 27) = 5.491, *P* < 0.05] and no significant effects of the “Genotype” [F(1, 27) < 1] or of the “Ceftriaxone” [F(1, 27) = 2.339, *P* > 0.05] factors on the mRNA levels of *Bace1* in the hypothalamus of the rats. LSD post hoc test revealed that the parameter was significantly lower in the rats of “OXYS + CEF” group versus that in rats of “OXYS + saline” (*P* < 0.05) group.

Due to the lack of normal distribution of the data in the studied groups, *Kruskal*–*Wallis ANOVA by Ranks* was applied and revealed no significant differences between the groups for mRNA levels of *Ece1* [H(3, 33) = 2.429, *P* > 0.05], *Epo* [H(3, 32) = 1.726, *P* > 0.05], and *Ide* [H(3, 34) = 1.461, *P* > 0.05]. For *Mme*, *Kruskal*–*Wallis ANOVA by Ranks* showed a significant difference between the groups: [H(3, 33) = 8.304, *P* = 0.040]. However, multiple comparisons of mean ranks for all groups demonstrated no significant intergroup differences.

#### *Actb*

Two-way ANOVA did not show a significant effect of the “Genotype” [F(1, 29) < 1] or “Ceftriaxone” [F(1, 29) = 2.070, *P* > 0.05] factor as well as of the interaction between the factors [F(1, 29) < 1] on the mRNA levels of *Actb*.

### Analysis of mRNA levels of CEF target genes in the hippocampus

**Fig. 3:**
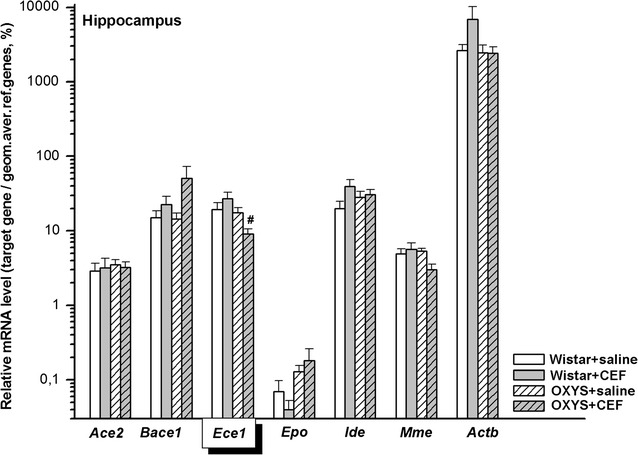
Effects of CEF on mRNA levels of *Ace2*, *Bace1*, *Ece1*, *Epo*, *Ide*, *Mme*, and *Actb* in the hippocampus of OXYS and Wistar rats. The data represent the relative mRNA levels of target genes to geometrical mean of *B2m*, *Hprt1*, and *Mapk6* mRNA levels. The data are expressed as the Mean ± S.E.M. of the values obtained in an independent group of animals (*n* = 6–9 per group). Statistically significant differences: ^#^*P* < 0.05 versus “Wistar + CEF” group

Abundance of mRNA species in the hippocampus is summarized in Fig. 3.

Due to the lack of normal distribution of the data in the studied groups, *Kruskal*–*Wallis ANOVA by Ranks* was applied and revealed no significant differences between the groups for mRNA levels of *Ace2* [H(3, 30) = 1.657, *P* > 0.05], *Bace1* [H(3, 34) = 2.317, *P* > 0.05], *Epo* [H(3, 22) = 5.239, *P* > 0.05], and *Actb* [H(3, 32) = 1.433, *P* > 0.05].

#### *Ece1*

According to two-way ANOVA, there was a significant influence of the “Genotype” factor [F(1, 27) = 5.462, *P* < 0.05], a tendency for the interaction between the factors [F(1, 27) = 3.511, *P* = 0.072] and no significant effect of the “Ceftriaxone” factor [F(1, 27) < 1] on the mRNA levels of *Ece1* in the hippocampus of the rats. LSD post hoc test revealed that the parameter was significantly lower in the rats of “OXYS + CEF” group versus that in rats of “Wistar + CEF” group (*P* < 0.01).

#### *Ide*

Two-way ANOVA did not show a significant effect of the “Genotype” [F(1, 29) < 1] or “Ceftriaxone” [F(1, 29) = 2.858, *P* > 0.05] factor as well as of the interaction between the factors [F(1, 29) = 1.677, *P* > 0.05].

#### *Mme*

According to two-way ANOVA, there was a tendency for influence of the interaction between the factors [F(1, 27) = 3.261, *P* = 0.082] and no significant effect of the “Genotype” factor [F(1, 27) = 1.785, *P* > 0.05] or of the “Ceftriaxone” factor [F(1, 27) < 1] on the mRNA levels of *Mme* in the hippocampus of the rats.

### Analysis of mRNA levels of CEF target genes in the striatum

**Fig. 4:**
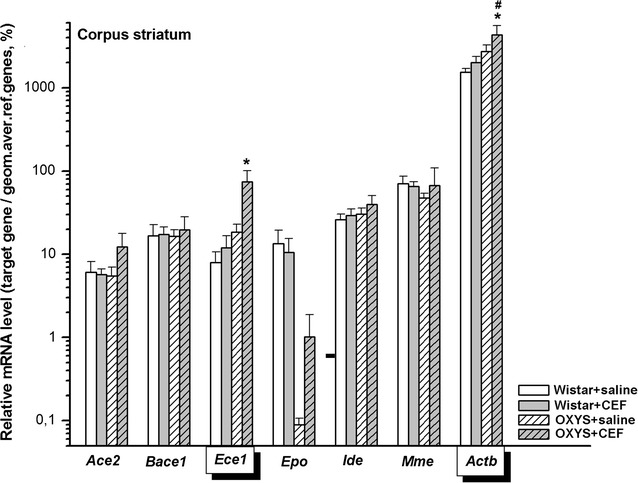
Effects of CEF on mRNA levels of *Ace2*, *Bace1*, *Ece1*, *Epo*, *Ide*, *Mme*, and *Actb* in the striatum of OXYS and Wistar rats. The data represent the relative mRNA levels of target genes to geometrical mean of *B2m*, *Hprt1*, and *Mapk6* mRNA levels. The data are expressed as the Mean ± S.E.M. of the values obtained in an independent group of animals (*n* = 6–9 per group). Statistically significant differences: *P < 0.05 versus “Wistar + saline” group; ^#^*P* < 0.05 versus “Wistar + CEF” group

Abundance of mRNA species in the striatum is summarized in Fig. 4.

Due to the lack of normal distribution of the data in the studied groups, *Kruskal*–*Wallis ANOVA by Ranks* was applied and revealed no significant differences between the groups for mRNA levels of *Ace2* [H(3, 30) = 2.069, *P* > 0.05], *Bace1* [H(3, 30) = 0.623, *P* > 0.05], *Epo* [H(3, 27) = 2.162, *P* > 0.05], and *Mme* [H(3, 31) = 2.085, *P* > 0.05]. For *Ece1*, *Kruskal*–*Wallis ANOVA by Ranks* showed a significant difference between the groups [H(3, 31) = 8.654, *P* = 0.034]. Multiple comparisons of mean ranks for all groups revealed that the parameter was significantly higher in the rats of “OXYS + CEF” group versus that in rats of “Wistar + saline” group (*P* < 0.05).

#### *Ide*

Two-way ANOVA did not show a significant effect of the “Genotype” [F(1, 29) = 1.385, *P* > 0.05] or “Ceftriaxone” [F(1, 29) < 1] factor as well as of the interaction between the factors [F(1, 29) < 1].

#### *Actb*

According to two-way ANOVA, there was a significant influence of the “Genotype” factor [F(1, 25) = 7.084, *P* < 0.05], and no significant effects of the “Ceftriaxone” factor [F(1, 25) = 2.401, *P* > 0.05] and of the interaction between the factors [F(1, 25) < 1] on the mRNA levels of *Actb* in the striatum of the rats. LSD post hoc test revealed that the parameter was significantly higher in the rats of “OXYS + CEF” group versus that in Wistar rats of “Wistar + saline” (*P* < 0.05) or “Wistar + CEF” (*P* < 0.05) group.

### Analysis of mRNA levels of CEF target genes in the frontal cortex

**Fig. 5:**
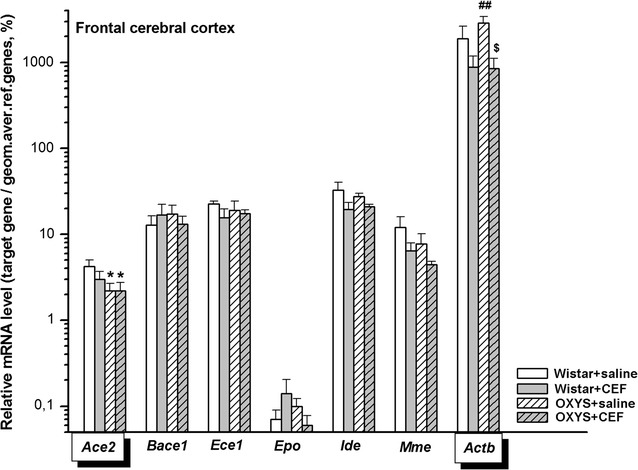
Effects of CEF on mRNA levels of *Ace2*, *Bace1*, *Ece1*, *Epo*, *Ide*, *Mme*, and *Actb* in the frontal cerebral cortex of OXYS and Wistar rats. The data represent the relative mRNA levels of target genes to geometrical mean of *B2m*, *Hprt1*, and *Mapk6* mRNA levels. The data are expressed as the Mean ± S.E.M. of the values obtained in an independent group of animals (*n* = 6–9 per group). Statistically significant differences: *P < 0.05 versus “Wistar + saline” group; ^##^*P* < 0.01 versus “Wistar + CEF” group; ^$^*P* < 0.05 versus “OXYS + saline” group

The results on the mRNA abundance in the frontal cortex are summarized in Fig. 5.

#### *Ace2*

According to two-way ANOVA, there was a significant influence of the “Genotype” factor [F(1, 24) = 4.416, *P* < 0.05], and no significant effect of the “Ceftriaxone” factor [F(1, 25) < 1] or of the interaction between the factors [F(1, 25) < 1] on the mRNA levels of *Ace2* in the frontal cortex of the rats. LSD post hoc test revealed that the parameter was significantly higher in the rats of “Wistar + saline” group versus that in rats of “OXYS + saline” (*P* < 0.05) or “OXYS + CEF” (*P* < 0.05) group.

#### *Bace1*

Due to the lack of normal distribution of the data in the studied groups, *Kruskal*–*Wallis ANOVA by Ranks* was applied and revealed no significant differences between the groups [H(3, 30) = 0.780, *P* > 0.05].

#### *Ece1*

Two-way ANOVA did not show a significant effect of the “Genotype” [F(1, 26) < 1] or “Ceftriaxone” [F(1, 26) = 1.587, *P* > 0.05] factor as well as of the interaction between the factors [F(1, 26) < 1].

#### *Epo*

According to two-way ANOVA, there was a tendency for influence of the interaction between the factors [F(1, 22) = 3.288, *P* = 0.083] and no significant effects of the “Genotype” [F(1, 22) < 1] and “Ceftriaxone” [F(1, 22) < 1] factors on the mRNA levels of *Epo* in the frontal cortex of the rats.

#### *Ide*

Two-way ANOVA did not show a significant effect of the “Genotype” [F(1, 22) < 1] or “Ceftriaxone” [F(1, 22) = 2.740, *P* > 0.05] factor as well as of the interaction between the factors [F(1, 22) < 1] on the mRNA levels of *Ide* in the frontal cortex of the rats.

#### *Mme*

Two-way ANOVA did not show a significant effect of the “Genotype” [F(1, 25) = 1.404, *P* > 0.05] or “Ceftriaxone” [F(1, 25) = 2.898, *P* > 0.05] factor as well as of the interaction between the factors [F(1, 25) < 1] on the mRNA levels of *Mme* in the frontal cortex of the rats.

#### *Actb*

According to two-way ANOVA, there was a significant influence of the “Ceftriaxone” factor [F(1, 21) = 9.455, *P* < 0.01], and no significant effect of the “Genotype” factor [F(1, 21) < 1] or of the interaction between the factors [F(1, 21) = 1.102, *P* > 0.05] on the mRNA levels of *Actb* in the frontal cortex of the rats. LSD post hoc test revealed that the parameter was significantly higher in the rats of “OXYS + saline” group versus that in Wistar rats of “Wistar + CEF” (*P* < 0.01) or “OXYS + CEF” (*P* < 0.05) group.

### Effects on body weight gain and biochemical parameters of plasma

We found no adverse side effects of CEF treatment on general condition and body weight gain of the animals. Body weight at sacrifice was 485.00 ± 9.81, 436.57 ± 18.18, 368.20 ± 18.49, or 364.60 ± 7.08 g in rats of “Wistar + saline”, “Wistar + CEF”, “OXYS + saline”, or “OXYS + CEF” group, respectively. Biochemical features of the groups are summarized in Table 1. While the levels of uric acid, creatinine, phosphate, and total bilirubin in plasma were not significantly affected by the factors, significant differences were revealed in the activity of ALT and AST, levels of Ca^2+^ and parameters of plasma lipid profile.

**Table 1:** Biochemical parameters of plasma in Wistar and OXYS rats treated chronically with CEF

Biochemical parameters	Wistar		OXYS	
	Control (saline)	CEF (100 mg/kg/day, 36 days)	Control (saline)	CEF (100 mg/kg/day, 36 days)
Uric acid (mmol/L)	7.48 ± 0.41	7.56 ± 0.26	7.70 ± 0.18	8.19 ± 0.42
Creatinine (µmol/L)	59.21 ± 0.44	57.70 ± 1.04	60.55 ± 0.71	58.74 ± 0.93
ALT, U/L	72.33 ± 4.03	70.56 ± 4.72	79.14 ± 2.58	64.00 ± 1.77^$^
AST, U/L	132.66 ± 3.34	137.64 ± 6.01	187.13 ± 15.04***^,###^	148.72 ± 4.51^$$^
Total bilirubin (µmol/L)	2.38 ± 0.21	1.99 ± 0.31	2.10 ± 0.21	1.79 ± 0.24
Ca^2+^ (mmol/L)	2.78 ± 0.03	2.70 ± 0.04	2.68 ± 0.03	2.59 ± 0.06**
Phosphate (mmol/L)	2.49 ± 0.06	2.46 ± 0.10	2.58 ± 0.17	2.30 ± 0.06
Total cholesterol (mmol/L)	1.82 ± 0.06	1.74 ± 0.14	1.42 ± 0.14*^,#^	1.56 ± 0.05
Triglycerides, g/L	1.07 ± 0.17	1.14 ± 0.12	1.30 ± 0.10	1.59 ± 0.15*^,#^
LDL-C (µmol/L)	0.463 ± 0.075	0.495 ± 0.089	0.213 ± 0.079*^,#^	0.200 ± 0.031*^,##^
HDL (mmol/L)	0.869 ± 0.042	0.843 ± 0.066	0.704 ± 0.060*	0.774 ± 0.045

Data are presented as the Mean ± S.E.M. of the values obtained in an independent group of animals (*n* = 7–8 per group)

Statistically significant differences: *P < 0.05, ***P < 0.001 versus “Wistar + saline” group; ^#^*P* < 0.05, ^##^*P* < 0.01, ^###^*P* < 0.001 versus “Wistar + CEF” group; ^$^*P* < 0.05, ^$$^*P* < 0.01 versus “OXYS + saline” group

Two-way ANOVA showed significant influence of the “Genotype” factor [F(1, 27) = 13.857, *P* < 0.001] and the interaction of the factors [F(1, 27) = 6.069, *P* < 0.05] on AST activity, and there was a tendency for the “Ceftriaxone” factor [F(1, 27) = 3.603, *P* = 0.068]. CEF treatment significantly reduced the augmented AST activity in OXYS rats. Similarly, chronic treatment with CEF attenuated the activity of ALT in OXYS rats. Two-way ANOVA revealed significant effect of the “Ceftriaxone” factor [F(1, 27) = 5.603, *P* < 0.05] and a tendency for the interaction of the factors [F(1, 27) = 3.510, *P* = 0.072] but not of “Genotype” factor [F(1, 27) < 1] on ALT activity. For the levels of Ca^2+^, two-way ANOVA showed significant influence of the “Genotype” [F(1, 27) = 7.14, *P* < 0.05] and “Ceftriaxone” [F(1, 27) = 4.36, *P* < 0.05] factors while the interaction of the factors was insignificant [F(1, 27) < 1]. LSD post hoc test revealed that the parameter was significantly lower in the rats of “OXYS + CEF” group versus that in Wistar rats of “Wistar + saline” (*P* < 0.01) group.

Significant influence of the “Genotype” factor was found on the levels of total cholesterol [F(1, 27) = 6.985, *P* < 0.05], triglycerides [F(1, 27) = 6.055, *P* < 0.05], LDL-C [F(1, 27) = 13.622, *P* < 0.001], and HDL [F(1, 27) = 4.483, *P* < 0.05] while the effects of “Ceftriaxone” factor or the interaction of the factors were insignificant.

## Discussion

In our laboratory, comparing Wistar to OXYS rats, we found that at the age of 5 months, OXYS rats demonstrated impaired novel object recognition and retarded learning in a T-maze test [14]. Furthermore, parallel studies illustrated that compared to Wistar, OXYS rats presented with age-related somatic alterations, early signs of mental decline similar to AD pathology, such as learning and memory deficits, gradual deterioration of cognitive function along with the neurodegenerative changes. At the age of 3 months, OXYS rats displayed significant associative learning deficits in a single-trial passive avoidance test [16]. Moreover, OXYS rats were characterized by a progressive decline in spatial learning and memory at the age of 3–16 m.o. in a Morris water maze [10, 13, 17]. Those cognitive deficits in OXYS rats occurred along with changes in the hippocampal synaptic plasticity including a deficit in long-term potentiation (LTP) [18] and demyelinating lesions detected using MRI starting at the age of 3 m.o. Furthermore, a 4% reduction in the brain volume was observed by the age of 12 m.o. [11]. Histological and immunohistochemical studies of young OXYS rats (3–5 m.o.) revealed neurodegenerative changes in the CA1, CA3, and dentate gyrus regions of the hippocampus including the increased percentage of dead or damaged neurons [15, 19, 20]. A trend of a decrease in the density of neuronal somas and fibers in the nigrostriatal DAergic system was also noted in OXYS rats [15]. At ultrastructural and molecular levels, young OXYS rats possessed mitochondrial and synaptic degeneration [12].

Additional studies illustrated marked extracellular and vascular Aβ deposits in the brain of OXYS rats that were detected at the age of 15–18 or 24 m.o., in rare cases—at the age of 3–7 m.o. Most brain structures (cerebral cortex, hippocampus, thalamus, hypothalamus, brain stem) except for the cerebellum were affected by Aβ plaque pathology, with the highest Aβ load in the cerebral cortex. Noteworthy that the levels of soluble oligomeric Aβ1–42 fractions, the most neurotoxic Aβ forms, were significantly augmented in OXYS rats at the age of 7, 12, and 24 m.o. [12]. Thus, one may conclude that Aβ metabolism is altered in OXYS rats and we may expect to find changes in the enzymes involved in Aβ production, degradation, or clearance in the rats even at young age.

Earlier, comparative SNP analyses disclosed the absence of specific for the early familial forms of AD mutations in *App*, *Psen1*, and *Psen2* genes in the OXYS genome [12]. At the same time, comparison of OXYS and Wistar rats by next-generation RNA sequencing revealed significant differences in gene expression of more than 900 genes at the age of 5 m.o. (and more than 2000 genes at the age of 18 m.o.) in the prefrontal cortex. Most of those genes were related to neuronal plasticity, protein phosphorylation, Ca^2+^ homeostasis, hypoxia, immune processes, and apoptosis. Among genes related to Aβ metabolism, mRNA levels of *Mme* encoding neprilysin (NEP) were decreased in the prefrontal cortex of OXYS with age and did not differ from Wistar rats at the age of 5 m.o. [21]. Here we also did not find significant differences in mRNA levels of *Mme* in the frontal cortex as well as other brain structures studied in 5-month-old OXYS rats as compared to Wistar controls. Noteworthy, mRNA levels of *Ece1* and *Ide* encoding other main amyloid-degrading enzymes, endothelin-converting enzyme (ECE)-1 and insulin-degrading enzyme (IDE), respectively, as well as of *Bace1* encoding β-secretase BACE1 did not vary significantly between control OXYS and Wistar rats. These results indicate that the expression of genes encoding the main enzymes of Aβ synthesis and degradation is not substantially affected (or compensated) in OXYS rats at young age and other mechanisms are likely to define the progression of neurodegenerative pathology at this stage.

Although some studies showed that angiotensin-converting enzyme (ACE) degrades Aβ42 to Aβ40 [22–24], overall data on the contribution of ACE to Aβ clearance are rather contradictory. Current data provide conflicting information on whether activation or inhibition of ACE could be beneficial in AD. Moreover, the efficiency of hydrolysis of Aβ by ACE is much inferior to NEP, IDE, and ECE and hence correlations between ACE levels and AD may relate more to effects on the renin-angiotensin system and brain vascular changes than to a major contribution to Aβ clearance [9]. Hence, we did not include *Ace* into the present study. However, we found a significant decrease in mRNA levels of *Ace2* encoding angiotensin-converting enzyme 2 (ACE2) in the frontal cortex and hypothalamus of control OXYS rats. These results are in a good agreement with previous findings demonstrating that ACE2 activity was significantly reduced in the brain of AD patients compared with age-matched controls and correlated inversely with levels of Aβ and phosphorylated tau [25], ACE2 activity also showed a tendency to decrease in the serum of AD patients compared with normal controls [24]. ACE2 is considered to influence Aβ metabolism as converting the longer and neurotoxic forms of Aβ (Aβ43) to the shorter, less toxic forms of Aβ [24] as well as ameliorating Aβ-induced inflammatory response [26]. Thus, we suggest that the disturbance in *Ace2* expression in the brain of OXYS may partially contribute to Aβ metabolism alterations in rats of this strain.

We also revealed that the expression levels of *Actb* encoding β-actin varied substantially between the experimental groups and brain structures in our experiment, hence we further analyzed *Actb* as a target gene. β-actin specifically controls cell growth and migration [27] as well as axon growth and collateral branch formation in neurons [28]. A significant decrease of *Actb* mRNA levels in the amygdala of OXYS rats may indicate the marked neurodegenerative changes at neurite level.

Here we also studied the potential effects of the antibiotic drug CEF, which has neuroprotective activity, on the modulation of mRNA levels of genes related to the system of Aβ metabolism in the brain. Recently, we found that CEF revised cognitive and neuronal deficits in OXYS rats [15]. In the present study we applied the dose of 100 mg/kg/day and treatment course of 36 daily drug injections that appeared to be effective for correction of both behavioral and neuronal deficits in OXYS rats. As in the previous study, we found no adverse side effects on general condition and body weight gain of the animals. Moreover, here we checked the effects of CEF on biochemical parameters of plasma. Similar to our previous results [14], interstrain differences were revealed in the activity of AST and ALT, levels of Ca^2+^, and parameters of plasma lipid profile. CEF did not have any significant effects on the indices of Wistar rats and normalized levels of AST and ALT in OXYS rats. The latter finding may indicate hepatoprotective properties of CEF.

The effects of CEF on AD-related pathology were also studied earlier using transgenic mouse models, namely, 3xTg-AD [29] and APPPS1 [30] strains. In middle aged 12-month-old 3xTg-AD mice, CEF rescued cognitive decline but had no pronounced effect on APP processing, overall Aβ species levels (except for the increase in Aβ40 levels in the CEF-treated mice), or plaque pathology. CEF neuroprotective effects were attributed to the restoration of synaptic proteins and inhibition of tau accumulation via attenuation of glutamatergic excitotoxicity induced by Aβ deposits [29]. The study by Hefendehl et al. [30] was focused on the pathological changes in glutamate dynamics in the immediate vicinity of Aβ deposits in APPPS1 transgenic mice and altered neuronal activity in such microenvironment that were corrected by CEF treatment. Noteworthy, both studies implied the models of advanced stages of AD-like pathology with highly expressed Aβ plaque pathology.

Here we found that CEF influenced mRNA levels of *Bace1*, *Mme*, *Ece1*, *Ide*, *Ace2*, *Epo*, and *Actb*. The following effects may contribute to neuroprotective properties of CEF as decreasing Aβ burden: CEF diminished *Bace1* mRNA levels in the hypothalamus, augmented *Ide* and *Mme* mRNA levels in the amygdala as well as the levels of *Ece1* in the striatum of OXYS rats. Moreover, CEF treatment increased *Epo* mRNA levels in the amygdala. Erythropoietin (EPO) encoded by *Epo* gene is a growth hormone and cytokine with wide range of biological activity. EPO and its receptors are found in the CNS of mammals, and are essential for neurodevelopment, adult neurogenesis, and neuroprotection. In animal models of neurodegenerative diseases including AD, neuroprotective and neuroregenerative mechanisms of EPO are related to inhibiting apoptosis, reducing oxidative stress and inflammation, promoting the angiogenesis and neurogenesis, and maintaining blood brain barrier (BBB) integrity [31]. The latter effect might be especially important mechanism in OXYS model due to neurovascular disturbances registered in OXYS rats [10, 32, 33]. Moreover, recent evidence suggests that cerebrovascular degeneration contributes to the pathogenesis of AD by causing faulty clearance of Aβ across the BBB [34]. It should be also noted that recently we revealed synergistic effect of combined treatment with CEF and exogenous EPO on neuroprotection and improvement in cognition in a MPTP-induced PD rat model [35].

Interestingly, these results showed that CEF had the most pronounced effect in the amygdala augmenting *Epo*, *Ide*, and *Mme* mRNA levels. Many studies have determined the main cognitive impairment in the preclinical phase of AD is episodic memory, in which a network of the hippocampus, entorhinal cortex, and amygdala are involved. In a recent study by Rasero et al. [36], the authors found progressive brain dysfunction using Diffusion-Tensor brain networks across severity stages in AD, namely, variations in connectivity patterns that start in a module network clearly associated with memory function (including part of the hippocampus, amygdala, entorhinal cortex, fusiform gyrus, inferior and middle temporal gyrus, parahippocampal gyrus and temporal pole) and later on, alterations were found widespread along the entire brain. One may suggest that CEF effects in the amygdala found in our study may be related to restoration of episodic memory in the novel object recognition test in OXYS rats [15].

At present, the CNS target responsible for CEF neuroprotective properties is unknown. Some studies have suggested possible interactors such as GFAP [37], alpha-synuclein [38], or just recently revealed high-affinity binding partner of CEF, metallo-β-lactamase domain-containing protein 1 (MBLAC1) [39], although further pathways and their contribution to CEF neuroprotection via modulation of gene and/or protein expression should be further investigated. On the other hand, it is generally assumed that the up-regulation of glutamate transporter EAAT2 in glial cells is responsible for CEF-mediated neuroprotection via its ability to reduce extracellular glutamate levels and subsequent excitotoxicity [40]. This effect was found to involve NF-κB-mediated *EAAT2* promoter activation [41]. Further studies revealed another mechanism of CEF neuroprotection against oxidative glutamate toxicity. It was related to robust induction of the expression of the glutamate/cystine exchanger, system xc− , through Nrf2/ARE transcriptional program in different neuronal or glial cell culture models [42]. The present study disclosed the novel targets for CEF action that are expressed in neuronal or/and glial (*Bace1*, *Mme*, *Ece1*, *Ide*, *Ace2*, and *Epo*) or endothelial (*Ece1*, *Ace2*) cells [2, 9, 43, 44]. In this study, most changes in mRNA levels after CEF treatment were registered in OXYS but not in Wistar rats thus emphasizing the relation of those effects to AD-like pathogenetic processes. The exact mechanisms through which CEF modulates the expression of those genes and their cross-talk with the known mechanisms of CEF neuroprotection as well as brain structure specificity of the effects need to be elucidated in further studies. We cannot exclude the interaction of CEF with epigenetic elements directly involved in the regulation of gene expression.

## Conclusion

The results of the study demonstrated a significant decrease in mRNA levels of *Ace2* in the frontal cortex and hypothalamus in control OXYS rats compared to control Wistar rats while mRNA levels of *Mme*, *Ece1*, and *Ide* encoding main amyloid-degrading enzymes, as well as of *Bace1* encoding β-secretase BACE1 in the brain structures studied did not vary significantly between control OXYS and Wistar rats at young age of 5 m.o. A profound reduction of *Actb* mRNA levels found in the amygdala of OXYS rats may indicate the marked neurodegenerative changes at neurite level.

The study on the potential effects of CEF in the modulation of mRNA levels of genes related to the system of Aβ metabolism in the brain revealed that CEF diminished *Bace1* mRNA levels in the hypothalamus, augmented *Ide*, *Mme*, and *Epo* mRNA levels in the amygdala as well as the levels of *Ece1* in the striatum. Thus, those findings disclosed novel targets for CEF action that might be involved into neuroprotective mechanisms at early, pre-plaque stages of AD-like pathology development.

## Methods

### Experimental animals

14-week-old male Wistar rats and OXYS male rats of the same age from The Federal Research Center “Institute of Cytology and Genetics”, Siberian Branch of the Russian Academy of Sciences (Novosibirsk, Russia) were used. Rats were housed in groups of five in acrylic cages (40 × 60 × 20 cm) in an animal room under standard conditions (a natural light–dark cycle (16 h light and 8 h dark), temperature: 18–22 °C, relative humidity: 50–60%, standard food and water ad libitum). Each animal was handled for 5 min/day on three consecutive days, before taking into experiment. Rats were divided into four experimental groups: Control (Saline-treated) Wistar males (“Wistar + saline” group, n = 10), Ceftriaxone-treated at a dose of 100 mg/kg Wistar males (“Wistar + CEF” group, n = 10), Control (Saline-treated) OXYS males (“OXYS + saline” group, n = 10), and Ceftriaxone-treated at a dose of 100 mg/kg OXYS males (“OXYS + CEF” group, n = 10).

### General procedures and drug administration

Ceftriaxone was purchased from Roche (Switzerland). Starting from the age of 14-week old (day 1), rats received 36 daily intraperitoneal (i.p.) injections of saline (1 mL/kg; “Wistar + saline” and “OXYS + saline” groups) or 36 daily i.p. injections of CEF (100 mg/kg/day). Rats were weighed weekly during the experiment to correct drug dosages. The rationale behind the CEF dosage (100 mg/kg/day) adopted in the current study was based on our recent study showing neuroprotective effects of CEF in correcting behavioral and neuronal deficits in OXYS rats [15].

On the day 37 the rats were sacrificed by exposure to CO_2_ and decapitation. Although certain influence of hypoxia on mRNA levels of some of the target genes were found earlier [45, 46], those effects were registered after prolonged hypoxic treatment (for at least 24 h). In our experiment, rats were sacrificed within 5 min after exposure to CO_2_. To our knowledge, no significant effects of hypoxia on the expression of the genes involved into the present study were observed after such a short period of exposure. Immediately after sacrifice, blood samples were taken for biochemical assays and brain structures (frontal cortex, hippocampus, amygdala, hypothalamus, and striatum) were rapidly dissected on ice, frozen in liquid nitrogen, and stored at − 70 °C until the total RNA extraction.

### Quantitative real-time PCR (qPCR)

The relative amount of target mRNA was measured by qPCR.

Sequences of specific primers for the reference genes (*Actb*, *B2m*, *Hprt1*, *Mapk6*) and target genes (*Ace2*, *Bace1*, *Ece1*, *Epo*, *Mme*, and *Ide*) were designed using the Primer 3 (NCBI) (Table 2). Specificity of the primers was checked by search of homologous sequences in rat Refseq mRNA database by means of the software PrimerBlast (https://www.ncbi.nlm.nih.gov/tools/primer-blast/). Each pair of primers was tested using RT-PCR with fluorescent SYBR Green dye on the cDNA matrix of known structure in the thermocycler CFX96 (Bio-Rad, USA) followed by determination of the melting temperature of the reaction product. The PCR product of each primer pair was analyzed in polyacrylamide gel electrophoresis as well to verify the quality and length of produced amplicons.

**Table 2:** Reference and target genes PCR primer sequences (5′–3′), amplicon size

Symbol	Sequence (5′–3′)		Amplicon size (bp)
*Reference genes*			
*Actb*	GTTCGCCATGGATGACGATATCG	CATCACACCCTGGTGCCTAG	139
*B2m*	TGCCATTCAGAAAACTCCCCA	GAGGTGGGTGGAACTGAGACA	104
*Hprt1*	ACAGGCCAGACTTTGTTGGA	TGGCTTTTCCACTTTCGCTG	123
*Mapk6*	CTCGATGAGTCGGAGAAGTCCC	TGTTGGCTGACAGGTGTCTCT	115
*Target genes*			
*Bace1*	GGACAACCTGAGGGGAAAGTC	GTACTGGACAGCTGCCTTTGGTA	168
*Ace2*	TCATTCGATATTACACAAGGACCA	AGCAACTTCTGCCCAGCTT	130
*Mme*	GCTGTGGGGAGGCTTTATGT	AATTGCCAGGGCCTTCTCTT	165
*Ece1*	TTAGCGGGAGGTGCATCCAAA	CCTCGGAGAGTGAGTCCACC	111
*Ide*	GGCCTGAGCTATGATCTCCA	GATCGCATGTACGCCTCTTTG	164
*Epo*	ATGGGGGTGCCCGAACG	TACCTCTCCAGAACGCGACT	122

Because of high variability, using just one reference gene should be avoided [47]. Four reference genes which are recommended by Qiagen (http://www.sabiosciences.com/rt_pcr_product/HTML/PARN-000Z.html#function) and other researchers [48, 49] were chosen as the most stable for the assessment of relative mRNA levels in rat brain structures. However, the values for the *Actb* in our experiment appeared to vary substantially between the experimental groups and brain structures, hence it was further analyzed as target gene. All the primers were purchased from Medigen Ltd. (Novosibirsk, Russia); exon spanning primer sets including large introns were used to eliminate the detection of residual genomic DNA.

Extraction of total RNA from fresh-frozen tissue was performed using RNeasy Mini kit in accordance with the manufacturer’s instructions (Qiagen, Germany). The amount of brain tissue of each sample was about 20 mg. The concentration of total RNA was determined using a NanoDrop 1000 microspectrophotometer (Nano-Drop Technologies, Wilmington, DE, USA). RNA sample quality was evaluated using the A260/A280 absorbance. The minimal RNA concentration was 24.6 ng/μl, the maximal—88.6 ng/μl. The amount of about 250 ng of total RNA was used in the further RT-PCR reactions.

The reverse transcriptase reaction volume was 30 µL. Composition of the reaction mixture for reverse transcription reaction included: 5 µL of RNA sample, 0.3 μM forward and reverse primers, 64 units of RNase inhibitor (RNasin^®^, Promega, USA), 160 units of reverse transcriptase (Biosan, Russia), 0.8 mM dNTP (Medigen, Russia), 1× RT Buffer (Biosan, Russia), 11.6 µL H_2_O. The protocol of reverse transcription consisted of 3 stages: 25 °C for 5 min, 42 °C for 30 min, and 95 °C for 5 min.

Polymerase chain reaction was carried out in 25 µL of reaction mixture. Reaction mixture contained: 0.2 mM dNTP (Medigen, Russia), 2.5 mM MgCl_2_ (Medigen, Russia), 0.04 μM forward and reverse primers, 1/500 V (V-reaction volume) SYBR Green I dye (Sigma-Aldrich, USA), 1x buffer for Taq DNA polymerase (Medigen, Russia), 1 unit of Smart Taq DNA polymerase (Medigen, Russia), 5 µL of cDNA. Amplification was started by an initial activation of the enzyme at 95 °C for 180 s. Each amplification cycle included denaturation at 95 °C for 10 s, annealing at 58 °C for 30 s, and elongation at 72 °C for 20 s with fluorescence signal acquisition. The maximum cycle threshold (Ct) value was set at 40.

Raw data were exported from the thermocycler CFX96 and the quantitative analysis of the qRT-PCR data was performed according to Ruijter et al. [50] to calculate the starting concentration (N_0_) of each cDNA template. Due to high variability of *Actb* mRNA values, levels of the target genes were normalized to the geometric average of three reference gene mRNA levels (*B2m*, *Hprt1*, *Mapk6).*

### Biochemical assays

Trunk blood of a rat was collected into heparinized Green Vac-Tubes (Green Cross MS Corp., Korea) right after sacrifice, centrifuged for 20 min at 3000 rpm at + 4 °C, plasma was stored at − 24 °C until assay. Plasma levels of the uric acid, creatinine, calcium, phosphate, total cholesterol, low-density lipoprotein cholesterol (LDL-C), triglycerides and high-density lipoproteins (HDL) as well as the plasma activity of aminotransferases [aspartate aminotransferase (AST), alanine aminotransferase (ALT)] and the levels of total bilirubin were measured using clinical chemistry analyzer Konelab 30i and Konelab kits (Thermo Fisher Scientific Inc., USA) according to the manufacturer’s instructions [14].

### Data analysis

All results were presented as mean ± SEM and compared using two-way ANOVA followed by post hoc Fisher LSD test or nonparametric Kruskal–Wallis ANOVA followed by multiple comparisons of mean ranks for all groups (in case of the lack of normal distribution of the data in the studied groups). The independent variables for two-way ANOVA were Genotype (Wistar or OXYS strain) and Treatment (Saline or CEF). The level of significance was defined as *P* < 0.05. STATISTICA 10.0 software was used to perform all the statistical analyses.

## Abbreviations

Aβ: amyloid-beta
ACE: angiotensin-converting enzyme
*Ace*: gene of ACE
ACE2: angiotensin-converting enzyme 2
*Ace2*: gene of ACE2
AD: Alzheimer’s disease
*Aktb*: gene of β-actin
ALT: alanine aminotransferase
APP: amyloid precursor protein
*App*: gene of APP
ARE: antioxidant response element
AST: aspartate aminotransferase
*B2m*: gene of beta-2 microglobulin
BACE1: β-secretase
*Bace1*: gene of BACE1
BBB: blood brain barrier
CEF: ceftriaxone
CNS: central nervous system
EAAT2: excitatory amino acid transporter 2
ECE1: endothelin-converting enzyme 1
*Ece1*: gene of ECE1
EPO: erythropoietin
*Epo*: gene of EPO
GFAP: glial fibrillary acidic protein
HDL: high-density lipoproteins
*Hprt1*: gene of hypoxanthine phosphoribosyltransferase 1
IDE: insulin-degrading enzyme
*Ide*: gene of IDE
i.p.: intraperitoneal
LDL-C: low-density lipoprotein cholesterol
LTP: long-term potentiation
*Mapk6*: gene of mitogen-activated protein kinase 6
MBLAC1: metallo-β-lactamase domain-containing protein 1
MPTP: 1-methyl-4-phenyl-1,2,3,6-tetrahydropyridine
*Mme*: gene of membrane metalloendopeptidase
NEP: neprilysin
NF-κB: nuclear factor kappa B
Nrf2: nuclear factor erythroid 2-related factor 2
*Psen1*: gene of presenilin 1
*Psen2*: gene of presenilin 2
